# Complete chloroplast genome of *Acer tegmentosum* and phylogenetic analysis

**DOI:** 10.1080/23802359.2019.1640646

**Published:** 2019-07-18

**Authors:** Sang-Chul Kim, Sookyung Shin, Min-Woo Lee, Jei-Wan Lee

**Affiliations:** Department of Forest Bio Resources, National Institute of Forest Science, Suwon-si, Korea

**Keywords:** *Acer tegmentosum*, Sapindaceae, complete plastid genome, phylogenetic analysis

## Abstract

The aim of the present study was to sequence and analyze the complete plastid genome (i.e. plastome) of *Acer tegmentosum* Maxim. The plastome of *A. tegmentosum* was 156,435 bp in length and included both large (86,265 bp) and small (18,102 bp) single-copy regions, which were separated by a pair of identical inverted repeats (26,034 bp). The plastome contained 77 unique protein-coding genes, 30 tRNA genes, and four rRNA genes. In addition, the gene order and organization of the *A. tegmentosum* plastome were consistent with those of plastomes from other members of the Sapindaceae, and the overall GC content of the plastome was 37.8%. A phylogenetic tree that was based on 76 protein-coding genes demonstrated a sister relationship within genus *Acer.*

*Acer* L. (Sapindaceae) species are some of the most important trees in the Northern Hemisphere, particularly in the temperate regions of eastern Asia, eastern North America, and Europe (van Gelderen et al. 1994). In particular, *Acer tegmentosum* Maxim. is a deciduous broad-leaved species that is only distributed in the mountains of northern and central Korea, namely in Manchuria, the Amur region, and the Uchida River basin (Kim 1995). The species is recognized for its antioxidant activity and beneficial effects on liver disease; however, its existence is threatened by rapid deforestation (Hong et al. 2007). Therefore, there is an urgent need for the preparation of measures that will protect the species against extinction. The species, though, has not been included in any molecular studies, so it is necessary to develop genomic resources for *A. tegmentosum*, in order to obtain intragenic information that can be used both for the conservation of *A. tegmentosum* and to elucidate the evolution of the genus. Sequencing the plastid genome (plastome) will contribute to the development of a protection strategy for the species.

Fresh leaves were collected from Odaesan National Park, Gangwon-do, Korea (37°47′N, 128°34′E), and genomic DNA was isolated from fresh leaves using a Plasmid SV mini kit (GeneAll, Seoul, Korea). Extracted DNA were stored in the Plant DNA Bank of the National Institute of Forest Science (No. 0335132681; Suwon, Korea). Whole genome sequencing was performed using the Ion Torrent sequencing platform (Life Technologies, Carlsbad, CA) and filtered sequences were assembled using the plastome of *Acer buergerianum* (GenBank accession number KX098452) as a reference sequence. The sequenced fragments were assembled using Geneious R10 (Biomatters, Ltd., Auckland, New Zealand; Kearse et al. 2012), and annotation was performed using both DOGMA (http://dogma.ccbb.utexas.edu/) and BLAST searches. The tRNA genes identified using Geneious were validated using the web-based tool tRNAScan-SE (Schattner et al. 2005), with default settings. Finally, maximum likelihood (ML) tree searches and ML bootstrap searches were performed for a dataset of 76 protein-coding genes from 14 members of the Sapindaceae using the RAxML BlackBox web-server (https://raxml-ng.vital-it.ch/#/, Stamatakis et al. 2008). The RAxML analyses were run using rapid bootstrap analysis with a random starting tree and 100 ML bootstrap replicates.

The plastome of *A. tegmentosum* was double-stranded, circular, and 156,435 bp long, with two inverted repeat regions (IRs; 26,034 bp each) that were separated by large single-copy (LSC; 86,265 bp) and small single-copy (SSC; 18,102 bp) regions (GenBank accession number MK942342). The plastome contained 128 genes, including 83 protein-coding genes, 37 tRNA genes, and eight rRNA genes, and six, seven, and four of the protein-coding, tRNA, and rRNA genes, respectively, were duplicated in the IRs. In addition, the overall GC content of the plastome was 37.8% (LSC, 36%; SSC, 32.2%; IRs, 42.9%), and the monophyly of the genus was well-supported (100% bootstrap values, BS), with *A. tegmentosum* placed as sister to *A. morrisonense* (Figure 1). The *A. tegmentosum* plastome described here may contribute to a better understanding of the evolution of *Acer.*

**Figure 1.**
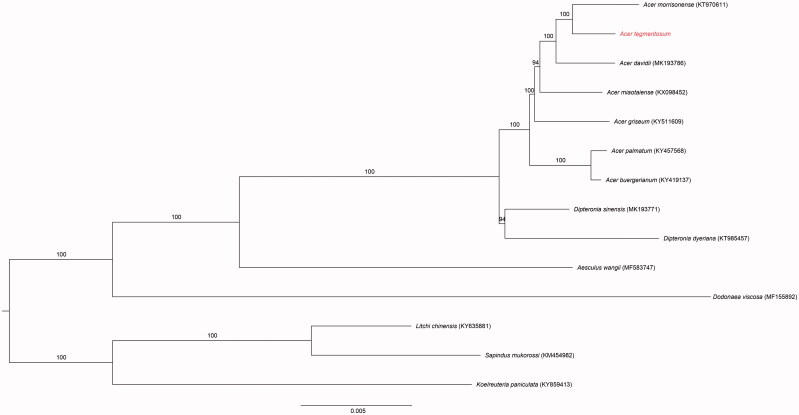
The maximum-likelihood (ML) tree based on the 14 representative chloroplast genomes of Sapindaceae. The bootstrap value based on 100 replicates is shown on each node.

## References

[CIT0001] Hong BK, Eom SH, Lee CO, Lee JW, Jeong JH, Kim JK, Kim MJ 2007 Biological activities and bioactive compounds in the extract of *Acer tegmentosum* Maxim. Stem. Korean J Med Crop Sci. 15:296–303.

[CIT0002] Kearse M, Moir R, Wilson A, Stones-Havas S, Cheung M, Sturrock S, Buxton S, Cooper A, Markowitz S, Duran C, et al. 2012 Geneious basic: an integrated and extendable desktop software platform for the organization and analysis of sequence data. Bioinformatics. 28:1647–1649. 22543367 10.1093/bioinformatics/bts199 PMC3371832

[CIT0003] Kim TW 1995 The woody plants of Korea in color. Seoul, Korea: Kyo-Hak Publishing Co, p. 476.

[CIT0004] Schattner P, Brooks AN, Lowe TM 2005 The tRNAscan-SE, snoscan and snoGPS web servers for the detection of tRNAs and snoRNAs. Nucleic Acids Res. 33:W686–W689. 15980563 10.1093/nar/gki366 PMC1160127

[CIT0005] Stamatakis A, Hoover P, Rougemont J 2008 A rapid bootstrap algorithm for the RAxML Web servers. Syst Biol. 57:758–771. 18853362 10.1080/10635150802429642

[CIT0006] van Gelderen DM, De Jong PC, Oterdoom HJ 1994 Maples of the world. Portland, Oregon: Timber Press.

